# An Integrated Prevention, Treatment, and Rehabilitation Program for Toileting-Related Falls in Older Adults and People with Disabilities: The Protocol for a Multicenter Prospective Single-Arm Implementation Study

**DOI:** 10.3390/healthcare14172794

**Published:** 2026-09-01

**Authors:** Zexuan Lv, Yin Lu, Hongyu Zhang, Rentang Li, Chengsong Mi, Kefeng Li, Fan Gao, Qiang Hu, Yi Wang, Qing Yuan

**Affiliations:** 1 Senior Department of Urology, Chinese PLA General Hospital, Beijing 100039, China; 2120241933@mail.nankai.edu.cn (Z.L.);; 2 School of Medicine, Nankai University, Tianjin 300071, China; 3 Chinese PLA Medical School, Beijing 100853, China

**Keywords:** toileting-related falls, fall prevention, disability, nocturia, rehabilitation, multidisciplinary care

## Abstract

**Background/Objectives**: Toileting-related falls may arise from interacting urinary symptoms, impaired mobility and balance, cognitive or psychological factors, environmental hazards, and caregiver limitations that are often managed separately. This study aims to evaluate the implementation, safety, and 6-month clinical outcomes of an integrated multidisciplinary prevention, treatment, and rehabilitation pathway for older adults and people with disabilities at risk of toileting-related falls. **Methods**: This multicenter, prospective, single-arm implementation study will enroll 300 adults aged ≥18 years across 15 centers. Participants will undergo standardized assessment of urinary function, gait and balance, physical function, psychological/cognitive factors, activities of daily living, fall history, and toileting-related environmental risks. Prespecified risk domain criteria will guide individualized urinary, rehabilitation, psychological/cognitive, caregiver, and environmental interventions, with reassessment and adjustment during follow-up. The primary outcome will be the 6-month cumulative incidence of at least one toileting-related fall. The secondary outcomes will include nighttime and total falls, urinary symptoms, nocturia, mobility, and functional independence. The implementation will be evaluated using RE-AIM and intervention fidelity measures. **Expected Results**: The study will characterize pathway reach, adoption, delivery, fidelity, safety, follow-up performance, data completeness, clinical event rates, and within-participant change. **Conclusions**: The findings may support a reproducible multidisciplinary care pathway and inform subsequent comparative evaluations of effectiveness, sustainability, component contributions, and resource use.

## 1. Introduction

Falls are a major public health problem and an important cause of injury, disability, loss of independence, and death in older adults. The burden is particularly high among people with frailty, disability, functional limitation, or dependence on caregivers. International guidance therefore recommends systematic risk identification, multifactorial assessment, tailored intervention, and longitudinal management rather than isolated single-component prevention strategies [1,2].

Toileting-related falls represent a distinct high-risk clinical scenario. They frequently occur during urgent or nighttime movement to and from the toilet, during transfer onto or off the toilet, or when a person mobilizes without sufficient assistance. Hospital studies show that many occur at night and that affected older adults often have overlapping cognitive impairment, urinary incontinence, gait instability, visual impairment, and dependence in daily activities [3,4]. These events are therefore better understood as the downstream result of interacting urinary, functional, cognitive, environmental, and care system vulnerabilities rather than as isolated environmental accidents.

Lower urinary tract symptoms can increase exposure to hazardous toileting situations. Urinary urgency, voiding difficulty, and nocturia are associated with incident or recurrent falls [5,6]. Systematic evidence also links overactive bladder and urgency urinary incontinence with increased risks of falls and fractures [7,8], supporting the need to consider urinary symptoms together with mobility and functional risk.

Fall prevention evidence supports multifactorial and multicomponent approaches when modifiable risks coexist [9,10]. Exercise targeting balance, gait, and muscle strength is a central component of fall prevention, and recent meta-analytic evidence further supports functional exercise for maintaining strength, functional capacity, and independence in older adults [11,12]. Environmental hazard reduction can also benefit high-risk community-dwelling older adults [13]. However, urinary symptom management, rehabilitation, psychological support, caregiver involvement, and environmental modification are often delivered in parallel rather than through a unified pathway, leaving interactions between these risks insufficiently addressed.

The present program is developed to address this gap by using the toileting scenario as a common endpoint of multiple interacting risks. It integrates urinary function, gait and balance, physical function, psychological/cognitive status, activities of daily living, environmental safety, and caregiver support within a closed-loop process of screening, multidimensional assessment, risk stratification, individualized intervention, follow-up, feedback, and dynamic adjustment. A theoretical model of lower urinary tract symptoms and falls similarly highlights urgency-driven movement, gait instability, divided attention, and executive dysfunction during toileting [14]. The study will evaluate its prospective implementation, safety, and clinical outcomes across participating centers. We hypothesize that a standardized risk-based pathway can be implemented consistently across different levels of care, as reflected by the prespecified RE-AIM and fidelity measures, while providing prospective estimates of 6-month toileting-related falls and changes in urinary, mobility, and functional outcomes. Because there is no concurrent control group, the clinical outcomes will be descriptive and hypothesis-generating rather than evidence of causal effectiveness.

## 2. Materials and Methods

### 2.1. Study Design and Setting

This multicenter, prospective, single-arm interventional implementation study will be conducted at 15 Chinese centers representing different levels of healthcare delivery: four tertiary Grade A hospitals, five secondary hospitals, and six primary-level hospitals. Chinese PLA General Hospital is the coordinating center.

The participating centers must have access to the target population; a designated local study team, the capacity to complete standardized multidimensional assessment and longitudinal follow-up; the ability to deliver or coordinate the core intervention domains; and the capacity to comply with common training, case report forms, fall surveillance, data quality, safety reporting, and ethical requirements. These minimum operational requirements are intended to preserve a common implementation standard across settings.

Including different levels of care is intentional and will allow for evaluation under real-world variations in case mix, staffing, rehabilitation capacity, equipment, and service organization. All centers will use the same eligibility criteria, assessment and intervention-assignment framework, outcome definitions, monthly fall surveillance schedule, documentation, training, and safety procedures. Specific intervention modalities may vary with participant-level clinical indications and local resources, but the underlying assessment and decision framework will remain standardized.

All participants will enter the same integrated pathway. There is no concurrent control arm, treatment allocation, or masking because the interventions are assigned based on a prespecified risk assessment and clinical indications.

**Figure 1:**
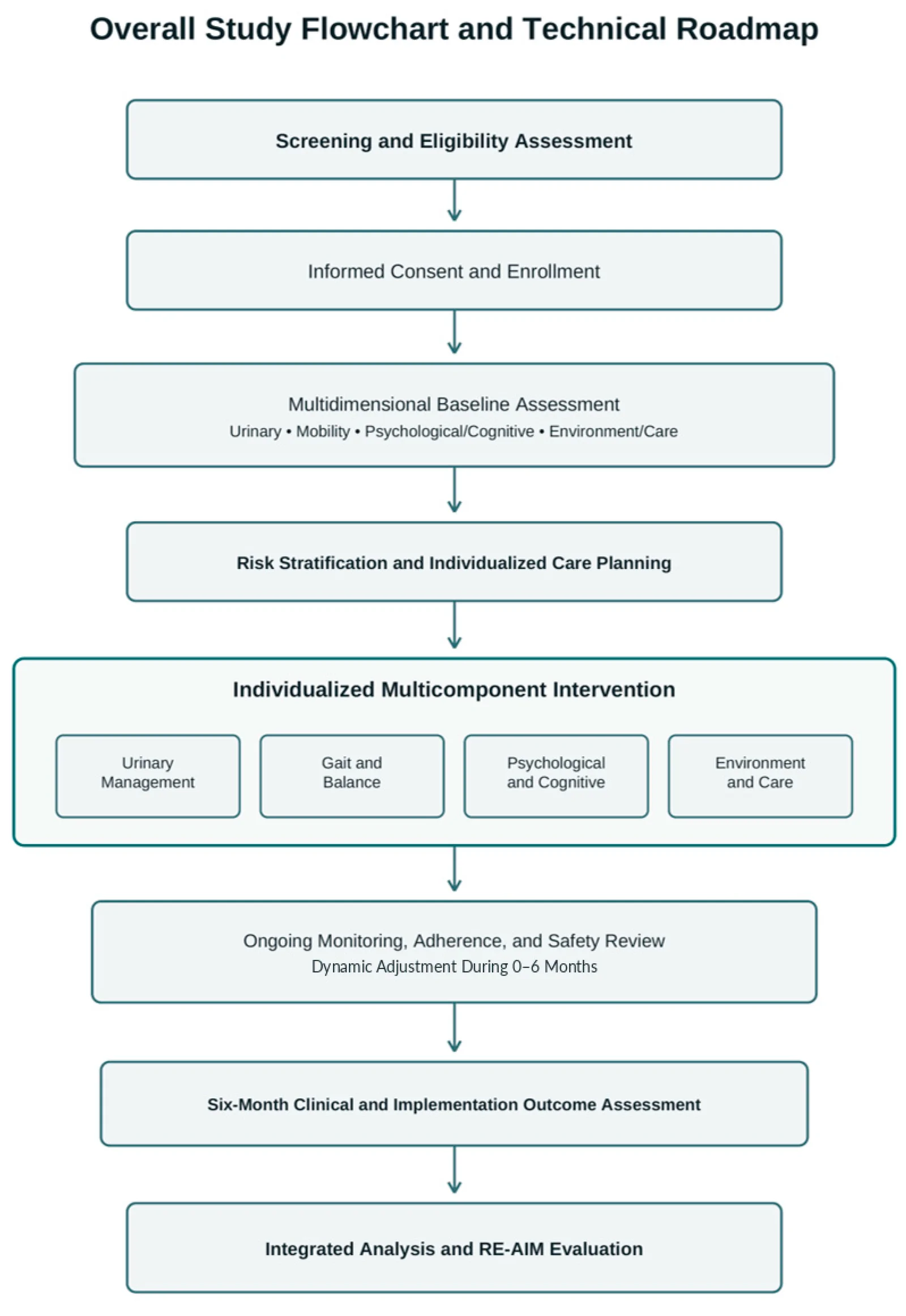
Overall study flowchart and technical roadmap.

The pathway comprises screening and consent, standardized baseline assessment; risk stratification; individualized multicomponent care; monitoring; reassessment; and adjustment according to response, adherence, safety, and changing risk. The follow-up will last 6 months (Figure 1; Table 1).

Figure 1 summarizes the screening, baseline assessment, risk stratification, individualized multicomponent intervention, monitoring and adjustment, 6-month outcome assessment, and integrated clinical and RE-AIM evaluation.

The protocol follows relevant prospective study-reporting principles and SPIRIT guidance [15,16].

### 2.2. Study Objectives

The primary clinical objective is to estimate the 6-month cumulative incidence of at least one toileting-related fall among participants managed through the pathway; this is an estimate of event occurrence, not causal effectiveness.

The secondary clinical objectives are to characterize nighttime and total falls and changes in urinary symptoms, nocturia, mobility, and functional independence. The implementation objectives are to assess the reach, adoption, delivery, fidelity, follow-up, data quality, and safety.

We hypothesize that the standardized pathway can be implemented consistently across levels of care, as assessed by the prespecified RE-AIM and fidelity measures. The clinical outcomes will be exploratory and will not be interpreted as confirmatory evidence of effectiveness.

### 2.3. Conceptual and Implementation Framework

The pathway assumes that toileting-related falls arise from interacting urinary, mobility/physical, psychological/cognitive, caregiver, and environmental risks rather than from a single disease process. These domains are assessed together so that intervention decisions target each participant’s dominant and modifiable risks and can be revised as those risks change.

Implementation evaluation will follow the RE-AIM framework [17]. The reach concerns entry of eligible individuals into the pathway; effectiveness comprises prespecified exploratory clinical and safety outcomes; adoption concerns center- and staff-level uptake; implementation concerns completion of assessment, delivery of risk-matched interventions, risk–intervention concordance, fidelity, documentation, and data completeness; and maintenance concerns 6-month follow-up completion and continued pathway delivery during the study period. Because the study is single-arm, the effectiveness outcomes will not be interpreted as comparative treatment effects.

Each RE-AIM domain is operationalized with prespecified measures, data sources, and time points. The design therefore combines clinical and implementation data while retaining the estimation-oriented, noncomparative nature of the protocol. Longer-term organizational maintenance beyond 6 months is outside the present study.

The RE-AIM definitions and measures are summarized in Table 2. Structured fall prevention programs, such as STEADI, support standardized training, workflows, risk assessment, and targeted intervention in routine care [18,19].

### 2.4. Participants

Eligible participants are adults at increased risk of toileting-related falls because of one or more interacting domains, including disability or functional limitation, loss of independence or care dependency, urinary symptoms, impaired mobility or balance, previous falls, cognitive or psychological problems, unsafe nighttime toileting, or environmental hazards.

The broad age range is intentional because the pathway is organized around a shared clinical problem—toileting-related fall risk—and common modifiable risk domains rather than a single diagnosis, disability type, or age group. Regardless of age, impairment profile, or recruitment setting, participants will undergo the same standardized baseline assessment, risk stratification process, outcome definitions, and follow-up procedures, while the intervention components will be individualized to identified risks. Prespecified subgroup analyses will explicitly characterize the age, functional, and setting-related heterogeneity.

Recruitment may occur in outpatient, inpatient, rehabilitation, long-term care, or community-linked services environments.

### 2.5. Eligibility Criteria

#### 2.5.1. Inclusion Criteria

1. Age 18 years or older, with no upper age limit.
2. Presence of physical disability, functional limitation, loss of independence, or a need for assistance with activities of daily living.
3. Presence of at least one risk factor for toileting-related falls, including but not limited to urinary frequency, urinary urgency, nocturia, urinary incontinence, voiding difficulty, overactive bladder, underactive bladder, impaired gait or balance, reduced muscle strength, fear of falling, cognitive or emotional problems, a history of falls, unsafe nighttime toileting, or toileting-related environmental hazards.
4. Ability and willingness to participate in study assessments and follow-up procedures, with caregiver assistance when necessary.
5. Written informed consent provided by the participant or, when applicable, a legally authorized representative.

#### 2.5.2. Exclusion Criteria

1. Acute infection or another acute medical condition requiring immediate treatment that would prevent participation in study assessments or interventions.
2. A severe or unstable medical condition that, in the investigator’s judgment, would make participation unsafe or interfere materially with study procedures or follow-up.
3. Severe disturbance of consciousness or another condition that would prevent completion of the required assessments and study procedures even with appropriate assistance.
4. Inability to participate in the planned follow-up or provide sufficient study-related information.
5. Any other condition that, in the investigator’s judgment, would make an individual unsuitable for participation.

### 2.6. Recruitment and Informed Consent

Recruitment is planned from 1 September to 31 December 2026 across all 15 centers. Investigators will explain study procedures, risks and benefits, alternatives, confidentiality, and voluntariness before obtaining written consent. When an individual’s capacity is insufficient, a legally authorized representative may consent in accordance with ethics requirements, with participant assent sought when feasible.

### 2.7. Multidimensional Baseline Assessment

The standardized baseline assessment will cover demographic characteristics, type and predominant profile of disability or functional impairment; comorbidities; recruitment setting; living arrangement; care dependency; previous falls; toileting-related circumstances; and the major urinary, mobility/physical, psychological/cognitive, caregiver, and environmental risk domains.

Key baseline covariates will be defined consistently across centers. Age will be recorded in completed years; recruitment setting as outpatient, inpatient, rehabilitation/long-term care, or community-linked services; baseline fall history as ≥1 fall in the previous 12 months; comorbidities as documented physician-diagnosed chronic conditions; and caregiver support as routine family, informal, or professional assistance with toileting, mobility, transfers, or activities of daily living. Baseline urinary symptom status and predominant disability/functional impairment profile will also be classified using standardized assessment.

Validated Chinese-language versions will be used for OABSS, PHQ-9, GAD-7, and FES-I, while POMA and MBI will follow standardized study procedures [20,21,22,23,24,25]. Because the cohort is clinically heterogeneous, each instrument will be administered only when its construct can be assessed validly according to prespecified applicability criteria. Protocol-defined non-administration will be documented separately from missing study data so that inapplicability is not treated as loss of information.

The urinary assessment will include symptom history, voiding records, nocturia frequency, urgency, urinary incontinence, voiding difficulty, and the clinically indicated PVR or Qmax. OABSS will be administered at baseline to participants with at least one storage symptom who can provide reliable self-reporting and be repeated at Month 6 when still applicable [20]. Participants with isolated voiding symptoms or an inability to provide reliable symptom self-reporting will not be required to complete OABSS; the reason for non-administration will be documented.

The physical assessment will include strength, joint mobility, transfers, sit-to-stand performance, gait, balance, mobility aids, and functional dependence. POMA will be performed at baseline and Month 6 in participants who can safely stand and ambulate, using habitual aids if needed; non-ambulatory or medically unsafe participants will not be tested [21]. MBI will assess activities-of-daily-living independence at baseline and Month 6 using participant, caregiver, and clinical information as appropriate [22].

The psychological/cognitive assessment will include fear of falling, anxiety, depressive symptoms, cognition, cooperation, and caregiver support. PHQ-9 and GAD-7 will be used at baseline when reliable self-reporting is possible [23,24]. FES-I will be used in participants able to self-report and who undertake standing, transfer, or ambulatory activities relevant to toileting [25]. Protocol-defined non-administration will be documented.

The environmental and care assessment will address nighttime lighting, bed-to-toilet route, floor hazards, thresholds, handrails, toilet height, bedside toileting devices, and caregiver response. The assessment–intervention–outcome matrix is shown in Table 3.

### 2.8. Risk Stratification and Intervention Assignment

After the baseline assessment, the multidisciplinary team will identify the clinically relevant and modifiable risks and assign one or more of five intervention domains: urinary, mobility/physical, psychological/cognitive, caregiver, and environmental. Each assignment requires a documented corresponding risk that materially contributes to toileting exposure, unsafe transfer or mobility, or fall risk; is potentially modifiable within the program; and has no clinical contraindication to the proposed intervention.

The domain-level assignment criteria will be standardized across centers, and assignment to one domain will not preclude simultaneous intervention in others. The specific modality, intensity, frequency, duration, and combinations may be tailored to symptom severity, functional capacity, safety, participant goals and preferences, caregiver capacity, and local resources. The planned intervention parameters and subsequent major modifications will be documented prospectively.

Urinary care will target symptoms that drive urgent, frequent, or nighttime toileting; rehabilitation will target deficits in strength, transfer, gait, balance, endurance, or mobility aid use; psychological/cognitive care will target factors that impair safe toileting or adherence; caregiver interventions will address assistance, supervision, or knowledge gaps; and environmental interventions will target modifiable hazards such as lighting, route obstacles, slippery surfaces, handrails, toilet height, and bedside facilities.

The CRF will document the identified risk factor, assigned intervention domain, selected component, clinical rationale, and subsequent modifications. When a potentially indicated component is not delivered because of contraindication, participant preference, caregiver limitation, or resource constraint, the reason will also be recorded. This documentation will support assessment of intervention exposure and risk–intervention concordance.

### 2.9. Intervention Components

To support reproducibility while retaining clinical individualization, each care plan will prospectively specify the intervention component; provider; setting; planned frequency; session duration or daily target when relevant; intended period; initial intensity or assistance; and criteria for progression, reduction, modification, or discontinuation. Actual exposure, adherence, major changes, and reasons for non-delivery or discontinuation will be recorded. The protocol standardizes this minimum specification rather than imposing one dose on a heterogeneous population, consistent with TIDieR principles (Table 4) [26].

#### 2.9.1. Urinary Symptoms and Nocturia Management

Urinary management may include fluid/voiding education, bladder training, scheduled voiding, medication review and optimization, and, when indicated, neuromodulation or electrical stimulation, with the aim of reducing unsafe toileting exposure.

#### 2.9.2. Gait, Balance, and Physical Rehabilitation

Rehabilitation may include strength, balance, gait, transfer, endurance, and targeted exercises plus adaptation of walking aids or wheelchairs. Exercise is a core fall prevention component [11,12].

#### 2.9.3. Psychological and Cognitive Support

Psychological/cognitive care may include psychological support, behavioral strategies, cognitive training, and reminders tailored to cognition, psychological status, function, and social support.

#### 2.9.4. Caregiver Support and Assisted Toileting

Caregiver interventions may include education, safe transfer and toileting instruction, reminders, assisted or scheduled toileting, and supervision during high-risk periods, tailored to dependency, cognition, toileting pattern, and caregiver capacity.

#### 2.9.5. Environmental and Toileting Safety Modification

Environmental care will match identified hazards with measures, such as improved lighting, obstacle or slip reduction, handrails/grab bars, toilet height adjustment, bedside toileting devices, or safer bed-to-toilet routes [13].

### 2.10. Training, Intervention Standardization, Fidelity Assessment, and Quality Assurance

Before implementation, the relevant staff will receive standardized training on eligibility assessment, multidimensional evaluation, risk stratification, intervention assignment and principles, device use when applicable, data collection, confidentiality, and safety reporting. Common manuals, CRFs, and intervention assignment checklists will be used across centers, and training completion will be documented for the personnel involved in assessment or intervention delivery.

Participant-level fidelity will assess completion of protocol-required and clinically applicable baseline assessments; documentation of identified risks and assigned domains; concordance between risks and assigned interventions; initiation of planned interventions or a documented reason for non-delivery; major modifications and their reasons; and completion of required follow-up and safety documentation. Concordance will quantify both whether assigned domains are supported by documented baseline risks and whether clinically relevant risks receive an intervention or a documented reason for non-delivery.

Center-level implementation consistency will be evaluated using assessment completion, risk–intervention concordance, intervention delivery and documentation, adherence to monthly fall surveillance, follow-up completion, data completeness, and protocol deviations. Indicators will be summarized separately for each center and, where informative, by center type.

The coordinating center will review these indicators periodically. Recurrent deficiencies will trigger clarification, targeted retraining, and corrective feedback while preserving the common core protocol and documentation standards.

Fidelity deviations will include unjustified omission of required assessment, intervention assignment without documented indication, omission of indicated intervention without explanation, or failure to document a major care plan modification.

Quality control will cover process fidelity, data quality, and safety through standardized CRFs, range/logic and missing data checks, staff competency and equipment checks, adverse event documentation, and escalation procedures.

### 2.11. Follow-Up, Fall Ascertainment, and Dynamic Adjustment

Participants will be followed for 6 months using a standardized fall surveillance schedule across all centers, with structured contacts at Months 1, 2, 3, 4, and 5 and a final assessment at Month 6. Contacts may be conducted in person, by telephone, or by video according to accessibility; however, the surveillance interval, core questions, fall definitions, and event documentation procedures will be identical across centers. Additional clinical contacts may occur according to need but will not replace the scheduled surveillance.

The 6-month period was selected a priori to balance clinically meaningful prospective observation with feasibility in a multicenter implementation study. It permits estimation of 6-month toileting-related fall occurrence together with short-term changes in urinary symptoms, nocturia, mobility, functional independence, fidelity, safety, and follow-up performance. The monthly structured surveillance is intended to reduce recall-related underreporting; the study is not designed to establish long-term preventive effectiveness, recurrent fall risk beyond follow-up, or long-term organizational sustainability.

At enrollment, participants and/or caregivers will be instructed to record and promptly report any fall during follow-up. At each monthly contact, investigators will use a standardized surveillance form to determine whether a fall occurred since the previous contact and will document the date; relationship to going to, using, transferring on or off, or returning from the toilet; nighttime occurrence; location and circumstances; resulting injury; and assistance available at the time.

A fall is an event in which a participant unintentionally comes to rest on the ground, floor, or another lower level. Toileting-related falls will use the prespecified primary outcome definition. Caregivers may assist in reporting when participants cannot report reliably. Events reported between scheduled contacts will be reconciled at the next contact to avoid duplication.

Missed monthly contacts will be completed as soon as feasible and documented. At Month 6, all fall records will undergo a final completeness and classification review.

Care plans may be modified for new risks, symptom change, poor adherence, adverse events, or changes in function or care environment; major modifications and reasons will be documented.

### 2.12. Outcome Measures

#### 2.12.1. Primary Outcome

The primary outcome will be the 6-month cumulative incidence of at least one toileting-related fall, defined as a fall while going to, using, transferring on/off, or returning from the toilet. It will be calculated as the proportion of participants with ≥1 such fall among those with an evaluable 6-month outcome status and interpreted as event occurrence rather than causal effectiveness (Table 5).

#### 2.12.2. Secondary Outcomes

1. Number of nighttime toileting-related falls up to 6 months.
2. Total number of falls from any cause up to 6 months.
3. Change in OABSS from baseline to Month 6 among participants with applicable evaluable paired measurements [20].
4. Change in average nocturia episodes per night from baseline to Month 6 using participant/caregiver voiding records.
5. Change in POMA score from baseline to Month 6 among participants eligible for the assessment [21].
6. Change in MBI from baseline to Month 6 [22].

#### 2.12.3. Prespecified RE-AIM Implementation and Safety Outcomes

1. Reach: Enrollment rate among eligible screened individuals at completion of recruitment.
2. Adoption: Proportion of centers completing initiation requirements and starting enrollment.
3. Adoption: Proportion of designated personnel completing standardized protocol training before study procedures.
4. Implementation: Completion rate of required and clinically applicable baseline assessments.
5. Implementation: Proportion receiving ≥1 documented risk-matched individualized intervention during follow-up.
6. Implementation: Risk–intervention concordance and predefined fidelity deviation frequency during follow-up.
7. Implementation: Completeness of required baseline, intervention, and follow-up data at Month 6.
8. Maintenance: Participant 6-month follow-up completion rate.
9. Maintenance: Proportion of centers continuing protocol-based pathway delivery and follow-up through the 6-month study period.
10. Safety: Proportion experiencing ≥1 adverse event judged as related or possibly related to a study intervention during follow-up.

### 2.13. Safety Assessment and Adverse Event Reporting

Safety will be monitored throughout follow-up. For each adverse event, investigators will record the type, onset, severity, duration, management, outcome, and relationship to study intervention. Serious events will be managed according to clinical need and reported as required; device-related events or deficiencies will also be documented.

Serious adverse events will be reported to the sponsor, ethics committee, and relevant institutional authorities in accordance with applicable requirements.

### 2.14. Sample Size and Enrollment Target

A total of 300 participants are planned to be enrolled, consistent with the registered target. This target refers to participants enrolled and does not imply that all 300 will necessarily have evaluable 6-month outcome data because some loss to follow-up is anticipated.

Because the study is primarily designed to evaluate implementation of a complex individualized pathway rather than superiority of a single treatment against a concurrent control group, no treatment-effect-based sample size calculation was used. The pragmatic target supports multicenter implementation and reasonably precise estimation of key proportions. For an evaluable sample of 300, a simple binomial approximation gives a maximum 95% confidence interval half-width of about 5.7 percentage points for a proportion near 50%, with narrower intervals farther from 50%.

No cluster design effect inflation was applied because this is not a cluster-randomized comparative trial and a reliable intraclass correlation is unavailable. Center clustering will instead be addressed in the prespecified analyses, with center-specific estimates reported descriptively.

With a 10%, 15%, or 20% loss to follow-up, approximately 270, 255, or 240 participants would remain evaluable, corresponding to maximum 95% confidence interval half-widths of about 6.0, 6.1, or 6.3 percentage points for a proportion near 50%.

The numbers enrolled, completing follow-up, and included in each analysis will be reported with reasons for loss or non-evaluability. Center and subgroup analyses will be descriptive, not separately powered tests of differential effectiveness.

### 2.15. Statistical Analysis

The analyses will follow a prespecified statistical analysis plan using R software (version 4.4.3; R Foundation for Statistical Computing, Vienna, Austria). The continuous variables will be summarized as mean (SD) or median (IQR) according to the distribution, and the categorical variables as n (%). The distributional assumptions for the continuous variables and change scores will be assessed by histogram and quantile–quantile plots, supplemented by a Shapiro–Wilk test. Effect estimates will be accompanied by 95% confidence intervals where appropriate. The clinical analyses will be exploratory because the single-arm design has no concurrent comparison group.

The primary outcome will be reported as a proportion with a 95% confidence interval. Participants with a documented toileting-related fall before loss to follow-up will remain event positive; those without a documented event who discontinue before completing the 6-month ascertainment will have an incomplete primary outcome status rather than be classified as event free.

Nighttime toileting-related and total recurrent falls will be summarized as counts and person-time rates. A negative binomial regression with the log follow-up time as an offset will be the primary event rate model. Adjusted exploratory models will include the center as a random intercept when stable; if sparse events or non-convergence prevent estimation, the observed counts and person-time rates with confidence intervals will be reported descriptively.

The continuous secondary outcomes with applicable paired baseline/Month 6 measurements will be summarized as the within-participant change with 95% confidence intervals and analyzed using paired t tests or Wilcoxon signed-rank tests according to the change-score distribution. Additional repeated observations will be analyzed with linear or generalized mixed-effects models accounting for the within-participant correlation.

Potential center effects will be examined explicitly. The primary cumulative incidence estimate and key implementation outcomes will first be reported for the overall cohort, with center-specific estimates and confidence intervals presented descriptively. When a clustering adjustment is required, the center will be incorporated as a random intercept if a stable estimation is possible. If not, it will be included as a fixed categorical effect where feasible; otherwise, center-specific descriptive estimates will be used. The center findings will characterize implementation heterogeneity and will not be formal rankings of institutional performance.

The implementation and fidelity indicators will also be summarized by center type to characterize the variation across levels of care, without inferring differential effectiveness.

Exploratory multivariable models will use covariates prespecified a priori on clinical grounds rather than data-driven stepwise selection: age, sex, baseline fall history, comorbidity burden, baseline functional status, caregiver support, recruitment setting, baseline urinary symptom status, and predominant disability/functional impairment profile. Model complexity will be limited according to sample size and available event counts to reduce overfitting; participating centers will be included as a random intercept when stable estimation is possible.

Prespecified subgroup analyses will examine age (18–64 vs. ≥65 years), predominant impairment profile (physical/mobility, cognitive, or mixed/multidomain), recruitment setting, baseline urinary symptoms, and functional dependency. Subgroup estimates will include 95% confidence intervals; regression-based heterogeneity analyses will be exploratory when data permit, with no claims of differential effectiveness.

The RE-AIM and intervention fidelity outcomes will be summarized according to their prespecified operational definitions using frequencies, proportions, and 95% confidence intervals where appropriate, overall and by center. The risk–intervention concordance, fidelity deviations, assessment completion, intervention delivery, data completeness, and follow-up completion will be reported transparently. Intervention exposure will be described by the intervention domains and components received, initiation and delivery, adherence where applicable, and major modifications during follow-up, both overall and, where informative, by center and relevant participant subgroups. Because intervention assignment and intensity will be individualized according to clinical indications, the exposure analyses will remain descriptive and will not be used to infer causal treatment effects.

Enrollment, follow-up completion, and evaluability will be reported separately with reasons for loss and missing data patterns. For the primary outcome, extreme-case sensitivity bounds will alternatively classify participants with incomplete 6-month status and no prior documented toileting-related fall as event positive or event negative. The continuous secondary outcomes will use available paired measurements with missingness reported.

No multiplicity adjustment is planned for exploratory clinical or subgroup analyses. Tests will be two-sided, with an emphasis on effect estimates and 95% confidence intervals.

### 2.16. Data Management and Confidentiality

Data will be collected on standardized CRFs and managed in a secure electronic data-capture system, such as REDCap, with controlled access, audit trails, and structured validation [27].

Each participant will have a unique study identifier; direct identifiers will be stored separately and access restricted to authorized personnel. Data review will include range/logic checks, missing data queries, and periodic cross-center completeness and consistency checks.

### 2.17. Patient and Public Involvement

Patients and the public were not formally involved in protocol design, conduct, reporting, or dissemination planning. Participant/caregiver feedback may inform operational refinements subject to appropriate review and approval.

### 2.18. Trial Status

The study is registered at ClinicalTrials.gov (NCT07738354), will enroll 300 participants across 15 centers from 1 September to 31 December 2026, and will follow each participant for 6 months. As of 1 August 2026, recruitment had not begun and the registry status was not yet recruiting.

## 3. Expected Results

The study is expected to determine whether the integrated pathway can be delivered consistently across participating settings and to quantify the enrollment, assessment completion, risk-matched intervention delivery, fidelity, follow-up completion, data completeness, and intervention-related adverse events. It will also provide prospective estimates of 6-month toileting-related fall occurrence and within-participant changes in urinary symptoms, nocturia, mobility, balance, and independence in activities of daily living. Because there is no concurrent control group, clinical changes will be interpreted as exploratory and hypothesis-generating rather than evidence of causal effectiveness.

## 4. Discussion

This protocol addresses toileting-related falls as a multidimensional clinical and care-delivery problem rather than as an isolated mobility event. By integrating urinary symptoms, gait and balance, physical function, psychological/cognitive factors, caregiver support, and environmental hazards within one decision pathway, the program is intended to reduce fragmentation between disciplines that commonly manage these risks separately. The approach is particularly relevant to people with disability or care dependency, in whom several modifiable risks may coexist and change over time. Recent longitudinal evidence from residential care also suggests that changes in mobility, supervision, activity, and care conditions can accompany changes in fall occurrence, reinforcing the importance of the care environment when interpreting fall risk [28].

A major strength of the study is its prospective multicenter implementation focus. Standardized assessment, risk stratification, intervention assignment criteria, documentation, training, and quality control procedures are combined with flexibility in the intervention package so that care can be adapted to individual clinical indications and local resources. The RE-AIM-informed evaluation will provide information not only on clinical events and functional changes but also on the reach, adoption, intervention delivery, fidelity, follow-up, data quality, and safety. Together with the center-level implementation measures, these data should help identify which elements of the pathway are reproducible across settings and which require local adaptation.

The study also has important limitations. The absence of a concurrent control group prevents causal attribution of changes in fall outcomes or functional measures to the integrated program. Observed within-participant changes may reflect the intervention, natural recovery or deterioration, regression to the mean, concurrent clinical care, or other time-related factors; clinical outcomes will therefore remain exploratory and hypothesis-generating. Participants will intentionally be heterogeneous in age, disability or functional impairment, comorbidity, urinary symptoms, mobility, care setting, caregiver support, and intervention exposure. The prespecified subgroup analyses will characterize this heterogeneity descriptively rather than confirm differential effects. In addition, participants may receive different combinations and intensities of urinary, rehabilitation, psychological/cognitive, caregiver, and environmental interventions; consequently, any observed clinical change cannot be reliably attributed to a single component of the pathway. Fall ascertainment may remain vulnerable to incomplete reporting despite the monthly structured surveillance, and not all symptom or functional instruments will be applicable to every participant. Variations in staffing, equipment, rehabilitation capacity, case mix, and local service organization may also contribute to inter-center differences in delivery. The common core protocol, standardized training and documentation, uniform surveillance, prespecified assignment criteria, and center-level fidelity monitoring are intended to limit this variability, but the center and center-type findings will remain descriptive rather than confirmatory.

If the integrated pathway demonstrates acceptable implementation, fidelity, safety, and follow-up performance, subsequent research should evaluate its effectiveness using comparative designs, such as pragmatic controlled or cluster-randomized studies and should examine the cost, resource use, sustainability, and the contribution of individual intervention components. The anticipated findings will be relevant to care delivered jointly by urology, rehabilitation medicine, nursing, psychology, geriatrics or primary care and community or long-term care services. Coordinated multidisciplinary implementation is therefore a central feature of the program rather than an ancillary component.

## 5. Conclusions

This multicenter protocol integrates urinary, rehabilitation, psychological/cognitive, caregiver, and environmental management within one toileting-related fall pathway. It will evaluate its implementation and safety while generating prospective clinical estimates to guide later comparative, sustainability, and cost-effectiveness studies.

## Figures and Tables

**Table 1:** Schedule of enrollment, intervention, and follow-up assessments.

Study Activity	Screening/Baseline	Intervention and Dynamic Management (0–6 Months)	Six-Month Assessment
Written informed consent	●		
Eligibility screening	●		
Demographic and medical history	●		
Multidimensional baseline assessment	●		
Risk stratification and individualized care plan	●	Review and adjustment	
Urinary symptom/nocturia management		As indicated	Review
Gait, balance, and physical rehabilitation		As indicated	Review
Psychological/cognitive support		As indicated	Review
Environmental and caregiver intervention		As indicated	Review
Fall and toileting-related fall surveillance		Ongoing	Final verification
Adherence and intervention delivery monitoring		Ongoing	●
Adverse event monitoring		Ongoing	●
OABSS	●		●
Nocturia episodes/voiding record	●	As clinically indicated	●
Tinetti Performance-Oriented Mobility Assessment	●		●
Modified Barthel Index	●		●
Implementation outcome assessment		Ongoing	●

Note: The symbol ● indicates a prespecified assessment or procedure at the corresponding study stage. The intervention components are selected according to individual risk factors and clinical indications; not every participant receives every component. OABSS, Overactive Bladder Symptom Score.

**Table 2:** Operationalization of the RE-AIM framework in the present study.

RE-AIM Domain	Operational Definition	Prespecified Measures	Data Source/Time Point
Reach	Extent to which eligible individuals enter the integrated care pathway	Enrollment rate: Number enrolled/number of eligible screened individuals; distribution of enrolled participants by recruitment setting and key demographic/clinical characteristics	Screening and enrollment logs; recruitment period
Effectiveness	Exploratory clinical and safety outcomes observed during participation in the pathway	Proportion experiencing ≥1 toileting-related fall; nighttime toileting-related falls; total falls; change in OABSS, nocturia episodes, Tinetti score, and Modified Barthel Index; intervention-related adverse events	Baseline, prospective event surveillance, and Month 6 assessment
Adoption	Uptake of the pathway by participating centers and designated clinical/research personnel	Proportion of participating centers completing study initiation requirements and initiating enrollment; proportion of designated personnel completing standardized protocol training	Center initiation records and training logs; before and during recruitment
Implementation	Extent to which the standardized assessment, intervention assignment, delivery, and documentation procedures are implemented as intended	Completion of clinically applicable baseline assessments; proportion receiving risk-matched intervention; risk–intervention concordance; protocol fidelity/deviation rate; intervention documentation; required data completeness	CRFs, intervention records, fidelity records, and coordinating-center review; baseline through Month 6
Maintenance	Continued participation in and use of the pathway during the prespecified 6-month study period	Participant 6-month follow-up completion rate; proportion of centers continuing protocol-based follow-up and pathway delivery for enrolled participants through completion of the study follow-up period	Follow-up records and center implementation records; Month 6

**Table 3:** Multidimensional risk assessment, intervention, and outcome matrix.

Risk Domain	Core Assessment	Potential Intervention Components	Prespecified Outcomes
Urinary function	OABSS; urinary frequency; urgency; nocturia; urinary incontinence; voiding difficulty; voiding diary; clinically indicated PVR and Qmax	Behavioral and bladder training; fluid and voiding management; scheduled toileting; clinically indicated medication optimization; neuromodulation or electrical stimulation when appropriate	Toileting-related falls; nighttime toileting-related falls; OABSS; nocturia episodes
Gait, balance, and physical function	Muscle strength and tone; joint mobility; sit-to-stand and transfer ability; gait; balance; Tinetti assessment; mobility aids	Balance and gait training; strength and endurance training; transfer training; assistive device adaptation; rehabilitation therapy	Total falls; Tinetti score; Modified Barthel Index
Psychological and cognitive factors	Fear of falling; anxiety; depressive symptoms; cognition; fall-related psychological responses; treatment cooperation; caregiver support	Psychological support; behavioral strategies; cognitive training; reminders; caregiver education and structured assistance	Adherence; functional independence; fall occurrence; clinically relevant symptom change
Environment and care	Nighttime lighting; bed-to-toilet route; slippery surfaces; thresholds; handrails; toilet height; bedside facilities; caregiver response	Environmental modification; handrails and night lighting; non-slip measures; bedside toileting devices; assisted or scheduled toileting; care-process optimization	Environmental risk correction; safe toileting; toileting-related falls

**Table 4:** TIDieR-informed specification of core intervention domains.

Intervention Domain	Core Components	Provider/Setting	Planned Dose Specification	Tailoring/Progression	Exposure/Fidelity
Urinary	Fluid/voiding education; bladder training; scheduled voiding; medication review; neuromodulation/electrical stimulation if indicated	Urology/clinical team; participating care setting	Frequency/schedule; session/device parameters when applicable; intended duration	Adjust to symptoms, response, safety, and goals	Initiation, adherence/exposure, modifications, non-delivery and reason
Rehabilitation	Strength, balance, gait, transfer, and endurance training; mobility aid adaptation	Rehabilitation or appropriately trained clinical staff	Frequency; session duration; intervention period; initial intensity/assistance	Progress, reduce, or modify by function, tolerance, response, and safety	Sessions/exposure, adherence, modifications and reasons
Psychological/cognitive	Psychological support; behavioral strategies; cognitive training; reminders	Appropriately trained clinical staff	Planned frequency and duration of contacts/training	Adapt to cognition, psychological status, cooperation, and function	Delivery, adherence when applicable, and major modifications
Caregiver	Education; transfer/toileting assistance; scheduled toileting; supervision	Study staff with participant caregiver	Training contacts; assistance/supervision schedule	Adapt to dependency, toileting pattern, cognition, and caregiver capacity	Training/delivery, adherence, and changes
Environmental	Lighting; route; handrails; non-slip measures; toilet height/bedside device adaptation	Multidisciplinary team, caregiver, or local service personnel	Required modification(s) and implementation timing	Match identified hazards, safety, and feasibility	Completion/non-completion and reason

**Table 5:** Prespecified clinical, implementation, and safety outcomes.

Outcome Category	Outcome Measure	Time Frame
Primary	The 6-month cumulative incidence of at least one toileting-related fall	Up to 6 months after enrollment
Secondary	Number of nighttime toileting-related falls	Up to 6 months after enrollment
Secondary	Total number of falls from any cause	Up to 6 months after enrollment
Secondary	Change in OABSS	Baseline and Month 6
Secondary	Change in average nocturia episodes per night	Baseline and Month 6
Secondary	Change in Tinetti Performance-Oriented Mobility Assessment score	Baseline and Month 6
Secondary	Change in Modified Barthel Index	Baseline and Month 6
Reach	Enrollment rate among eligible screened individuals	At completion of recruitment
Adoption	Proportion of participating centers completing study initiation requirements and initiating participant enrollment	During the recruitment period
Adoption	Proportion of designated study personnel completing standardized protocol training	Before participation in study assessment or intervention delivery
Implementation	Completion rate of protocol-required and clinically applicable multidimensional baseline assessments	At baseline
Implementation	Proportion of participants receiving at least one documented individualized intervention matched to identified risk factors and clinical indications	Up to 6 months after enrollment
Implementation	Risk–intervention concordance rate	During the 0–6-month intervention period
Implementation	Proportion of participants with ≥1 predefined intervention fidelity deviation	Up to 6 months after enrollment
Implementation	Completeness of protocol-required study data	At Month 6
Maintenance	Six-month participant follow-up completion rate	At Month 6
Maintenance	Proportion of participating centers continuing protocol-based pathway delivery and follow-up for enrolled participants through completion of the 6-month study period	At completion of the 6-month study follow-up period
Safety	Proportion experiencing intervention-related or possibly related adverse events	Up to 6 months after initiation of intervention

## Data Availability

Individual participant data are not planned to be shared publicly because public data sharing was not specified in the current informed consent and ethics-approved protocol. Aggregate and de-identified study results will be disseminated through scientific publications and presentations. Any future request for access to de-identified data will require separate review by the study investigators and compliance with applicable ethical, legal, and institutional requirements.

## Abbreviations

GAD-7, Generalized Anxiety Disorder-7; OABSS, Overactive Bladder Symptom Score; PHQ-9, Patient Health Questionnaire-9; PVR, post-void residual urine volume; Qmax, maximum urinary flow rate; RE-AIM, Reach, Effectiveness, Adoption, Implementation, and Maintenance; REDCap, Research Electronic Data Capture; SPIRIT, Standard Protocol Items: Recommendations for Interventional Trials.
